# Modeling of Electric Field and Dielectrophoretic Force in a Parallel-Plate Cell Separation Device with an Electrode Lid and Analytical Formulation Using Fourier Series

**DOI:** 10.3390/s25010185

**Published:** 2024-12-31

**Authors:** Daiki Nishikawa, Yoshinori Seki, Shigeru Tada

**Affiliations:** Department of Applied Physics, National Defense Academy, Hashirimizu 1-10-20, Yokosuka 239-0802, Kanagawa, Japan; em62032@nda.ac.jp (D.N.); ed22004@nda.ac.jp (Y.S.)

**Keywords:** dielectrophoresis, cell separation, microfluidic device, electric field analysis, Fourier series expansion, three-dimensional fluorescence imaging

## Abstract

Dielectrophoresis (DEP) cell separation technology is an effective means of separating target cells which are only marginally present in a wide variety of cells. To develop highly efficient cell separation devices, detailed analysis of the nonuniform electric field’s intensity distribution within the device is needed, as it affects separation performance. Here we analytically expressed the distributions of the electric field and DEP force in a parallel-plate cell separation DEP device by employing electrostatic analysis through the Fourier series method. The solution was approximated by extrapolating a novel approximate equation as a boundary condition for the potential between adjacent fingers of interdigitated electrodes and changing the underlying differential equation into a solvable form. The distributions of the potential and electric fields obtained by the analytical solution were compared with those from numerical simulations using finite element method software to verify their accuracy. As a result, it was found that the two agreed well, and the analytical solution was obtained with good accuracy. Three-dimensional fluorescence imaging analysis was performed using live non-tumorigenic human mammary (MCF10A) cells. The distribution of cell clusters adsorbed on the interdigitated electrodes was compared with the analytically obtained distribution of the DEP force, and the mechanism underlying cell adsorption on the electrode surface was discussed. Furthermore, parametric analysis using the width and spacing of these electrodes as variables revealed that spacing is crucial for determining DEP force. The results suggested that for cell separation devices using interdigitated electrodes, optimization by adjusting electrode spacing could significantly enhance device performance.

## 1. Introduction

In recent years, the development of a rapid and highly accurate method of separating and detecting specific rare cells from large numbers of diverse cell populations has become an urgent priority. For instance, the presence of circulating tumor cells (CTCs) in the blood increases as cancer progresses, underscoring the effectiveness of the rapid and accurate detection of CTCs for ultra-early cancer diagnosis. However, it is challenging to isolate and detect rare cells, including very early-stage CTCs, which exist in minute quantities in the blood. In light of these challenges, dielectrophoresis (DEP) technology has recently attracted attention as an effective means of separating and detecting rare cells [1,2,3,4].

DEP is a phenomenon in which dielectric microparticles move along the gradient of a nonuniform electric field, propelled by the induction of electrical forces (DEP forces) acting on the microparticles. This results from the interaction between the dipole moment induced in the microparticles and the nonuniform electric field. In particular, using DEP with an AC electric field for cell separation offers significant advantages; it allows the separation of biological particles such as cells, viruses, and DNA with high accuracy and is non-invasive to such particles because it does not require pretreatments such as fluorescent labeling [5,6]. Many label-free cell separation methods have been proposed that utilize differences in cell mass and size [7,8]. In contrast, DEP leverages differences in electrical properties, making it an excellent method for separating cells with similar mass and size. However, since DEP relies on localized electric field gradients, careful device design is essential when separating large quantities of cells with high accuracy. However, planar electrodes, which are widely used in DEP cell separation technology, have a limited effective volume for processing cell samples, posing a challenge in achieving highly efficient separation of large quantities of cells [9]. Along with the increasing size of cell separation devices and the optimization of the parallel-plate microfluidic channel structure, which has been the basis of the cell separation device structure, devices with various shapes of microfluidic channels and electrodes have been proposed to enable the highly efficient separation of large numbers of cells [10,11,12,13,14,15,16,17,18,19,20,21,22]. However, issues such as increased applied voltage due to larger and more complex device structures have raised concerns about the adverse effects stemming from the generation of Joule heat and strong AC electromagnetic fields on the physiological functions of cells [23]. Therefore, there is a need to develop devices capable of highly efficient cell separation even under low-voltage loading conditions. Since the magnitude and distribution of the DEP forces acting on cells depend heavily on the electric field distribution, the precise determination of the electric field distribution will enable the optimal design of the device’s internal structure, electrode arrangement, and electrode architecture. This, in turn, is expected to facilitate the development of highly efficient cell separation devices that can operate under low-voltage conditions.

Numerous studies have aimed to rigorously determine the electric field in microfluidic devices and the DEP force induced by nonuniform electric fields. A wide range of methods exist for obtaining electric field distributions. For example, analytical approaches have utilized Fourier series [12,24,25,26,27], Green’s functions [28,29], and Green’s theorem [30,31], while other approaches have been based on the principle of conformal mapping, including the Schwarz–Christoffel transform [32,33,34,35,36], or on numerical simulations using finite element methods (FEMs) [37,38,39,40,41].

In this study, we derived analytical solutions for the distribution of the AC electric field and DEP forces generated in a parallel-plate cell separation device that we proposed previously [22,42] by applying electrostatic analysis using the Fourier series method. The particular advantage of analytical methods over numerical simulation methods such as FEM is, first of all, that they provide accurate and stable solutions when determining the spatial distribution of the DEP force. Furthermore, parametric analysis can be performed quickly and inexpensively, making it easy to investigate in detail the effects of electrode geometry and configuration on the distributions of the electric field and the dielectrophoretic force. With these advantages, the analytical method is an extremely powerful tool for device optimization design. In modeling the proposed device, whose top plate (lid) also serves as an electrode, we need to solve a mixed boundary value problem comprising three Dirichlet boundary conditions and one Neumann boundary condition for the electric potential, excluding the periodic boundary condition [43]. However, this problem differs significantly from conventional devices that have either a glass top plate or open-top interdigitated electrodes, where exact solutions are attainable, highlighting the severe challenge of obtaining closed-form solutions [12,14,31,33,35,44]. Therefore, in this study, we extrapolated a newly proposed approximate equation to the potential between adjacent fingers of interdigitated electrodes, originally necessitating a Neumann boundary condition. This was replaced with the corresponding Dirichlet boundary condition to reformulate the underlying differential equation into a solvable form, thus enabling the derivation of an analytical solution. The accuracy of the distributions of the potential and electric fields was verified by comparing those obtained by the analytical solutions to the results of numerical simulations using commercially available FEM software. Three-dimensional (3D) fluorescence imaging analysis was subsequently performed using live non-tumorigenic human mammary cells (MCF10A). The analytical model was validated by comparing the distribution of live cell clusters adsorbed on the interdigitated electrode surface to the analytically obtained distribution of the DEP force. The mechanisms underlying cell adsorption and cell cluster formation at the interdigitated electrode surface were also discussed. Furthermore, a parametric analysis was performed using the electrode width and spacing of the interdigitated electrodes as variables, and the optimal electrode arrangement and architecture for highly efficient cell separation were explored and proposed.

## 2. Theory

### 2.1. Basic Principle of DEP

The DEP force induced in the cell subjected to a nonuniform AC electric field is given as follows [4]:

(1) FDEP=2π ϵl( dc 2)3Re(β) ∇|E|2

where $\epsilon_{l}$ is the electric permittivity of the solution, $d_{c}$ is the spherical cell diameter, ∇ is the Nabla operator, and E is the AC electric field vector. Re(β) denotes the real part of the Clausius–Mossotti (CM) factor:

$$\beta =\frac{\epsilon_{c}^{*}-\epsilon_{l}^{*}}{\;\epsilon_{c}^{*}+2\;\epsilon_{l}^{*}}$$

where $\epsilon_{c}$ is the electric permittivity of the cell. The symbol ‘∗’ represents the complex electric permittivity and is expressed as

$$\epsilon^{*}=\epsilon +\frac{\sigma }{\;j\omega \;}\;,\;\;j=\sqrt{-1}$$

where σ is the electric conductivity, and ω is the angular frequency of the AC electric field (=2πf; f is the electric field frequency). For the application of the single-shell model to live mammalian cells, β can be expressed as [45]

(2) $$\beta =\frac{\;\omega^{2}\left(\tau_{l}\;{\tau_{c}}^{*}-\tau_{c}\;{\tau_{l}}^{*}\right)+j\omega \left(\;{\tau_{l}}^{*}-\tau_{l}-{\tau_{c}}^{*}\right)-1\;}{\;\omega^{2}\left(2\tau_{l}\;{\tau_{c}}^{*}-\tau_{c}\;{\tau_{l}}^{*}\right)-j\omega \left(\;{\tau_{l}}^{*}-2\tau_{l}-{\tau_{c}}^{*}\right)-2\;}$$

where

$$\;{\tau_{c}}^{*}=\frac{\;c_{m}d_{c}\;}{\;\;2{\sigma_{c}}^{\prime }\;}\;,\;\;\tau_{c}=\frac{{\epsilon_{c}}^{\prime }}{\;{\sigma_{c}}^{\prime }\;}\;,\;\;\;{\tau_{l}}^{*}=\frac{\;c_{m}d_{c}\;}{\;2\sigma_{l}}\;,\;\;\tau_{l}=\frac{\;\epsilon_{l}\;}{\;\sigma_{l}}.$$

**Figure 1:**
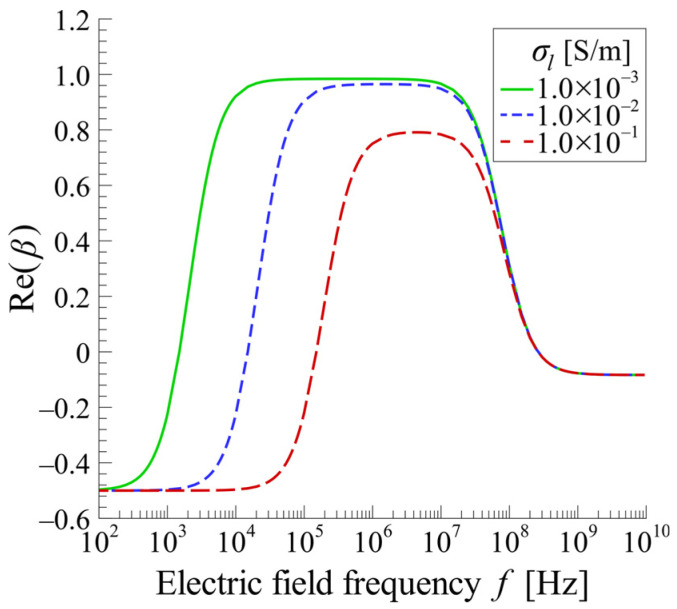
Frequency spectrum of Re(β) for various $\sigma_{l}$.

Here, $\sigma_{l}$ is the electric conductivity of the solution, ${\epsilon_{c}}^{\prime }$ is the effective electric permittivity of the cell, ${\sigma_{c}}^{\prime }$ is the effective electric conductivity of the cell, and $c_{m}$ is the capacitance of the cell membrane. As an example, Figure 1 shows the frequency spectra of the Re(β) of live MCF10A cells for various values of $\sigma_{l}$. Values of the $d_{c}$ and other electrical properties were taken from Ref. [46].

To evaluate the magnitude of $F_{\mathrm{DEP}}$ induced on the cell, it is necessary to know the values of Re(β) and $\nabla {\left|E\right|}^{2}$. The value of Re(β) can be obtained from Equation (2) if the values of $d_{c}$ and f are known, and the value of $\nabla {\left|E\right|}^{2}$ can be determined if the distribution of E can be obtained by some means.

### 2.2. Electric Field Analysis

**Figure 2:**
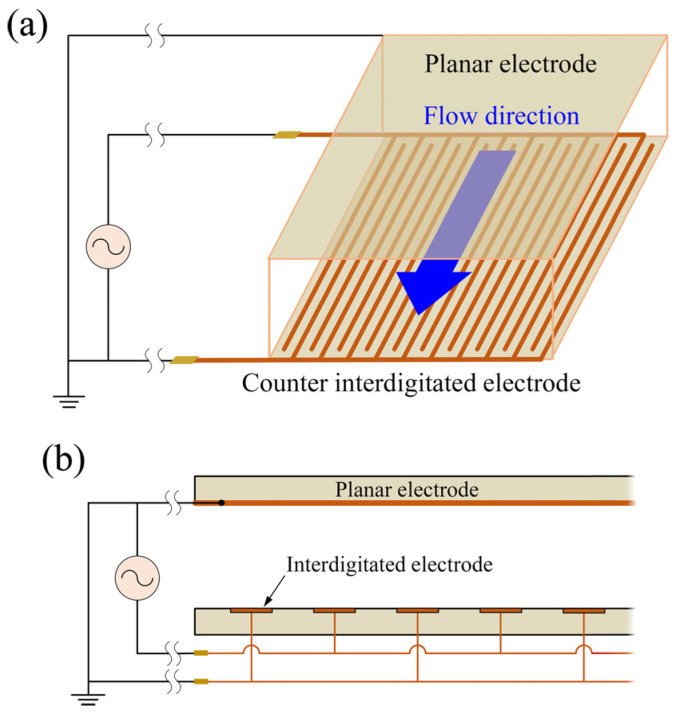
(**a**) Schematic of cell separation device. (**b**) Two-dimensional model of cell separation device.

Figure 2a shows a schematic of the cell separation device to be analyzed. The device has a parallel-plate microfluidic channel structure consisting of interdigitated electrodes on the bottom surface and a planar electrode on the top surface. By applying AC voltage to the device, a nonuniform electric field is generated across the whole volume of the microfluidic channel. Because the fingers of the interdigitated electrodes are sufficiently long and aligned along the flow direction of the microfluidic channel, the electric field analysis model in the device can be simplified into a two-dimensional (2D) model in the cross section of the microfluidic channel, as shown in Figure 2b.

**Figure 3:**
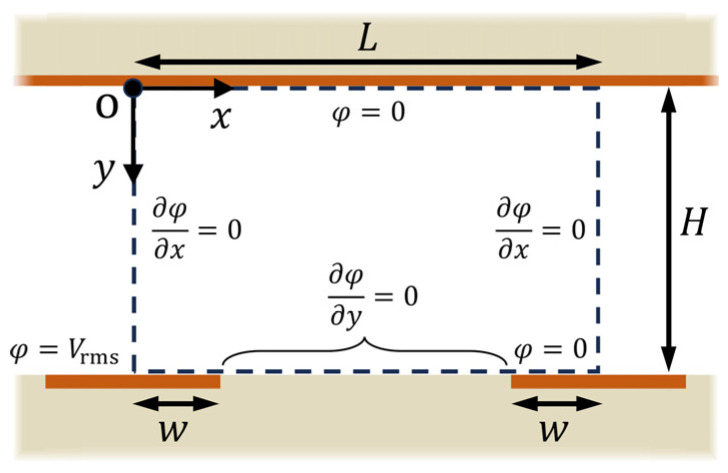
Mathematical model and boundary conditions.

The mathematical model of the electric field analysis is shown in Figure 3. In the present analysis, an electrostatic field analysis based on the AC effective value was adopted. The governing equation for the potential of the electrostatic field in the microfluidic channel, φ(x,y), is the Laplace equation.

(3) $$\frac{\;\partial^{2}\varphi \left(x,y\right)\;}{\partial x^{2}}+\frac{\;\partial^{2}\varphi \left(x,y\right)\;}{\partial y^{2}}=0.$$

The boundary conditions for the potential are

(4) $$\varphi \left(x,H\right)=V_{\mathrm{rms}}\;\;\;\;\;\;\left(0\leq x\leq w\right)$$

(5) $$\frac{\partial \varphi \left(x,H\right)}{\partial y}=0\;\;\;\;\;\;\left(w<x<L-w\right)$$

(6) φ(x,H)=0       (L−w≤x≤L)

(7) φ(x,0)=0

(8) $$\frac{\partial \varphi \left(0,y\right)}{\partial x}=0$$

(9) $$\frac{\partial \varphi \left(L,y\right)}{\partial x}=0$$

where $V_{\mathrm{rms}}$ is the effective value of the applied voltage, L is the distance between the center lines of adjacent interdigitated electrodes, H is the height of the microfluidic channel, and w is half the width of the electrode. Here, the following dimensionless variables are introduced.

(10) $$x^{*}=\frac{x}{\;L\;}\;\;\;$$

(11) $$y^{*}=\frac{y}{\;H\;}.$$

Substituting Equations (10) and (11) into Equations (3)–(9) yields

(12) $$\frac{\partial^{2}\varphi \left(x^{*},y^{*}\right)}{\partial x^{*2}}+{\left(\frac{L}{H}\right)}^{2}\frac{\partial^{2}\varphi \left(x^{*},y^{*}\right)}{\partial y^{*2}}=0$$

(13) $$\varphi \left(x^{*},1\right)=V_{\mathrm{rms}}\;\;\;\;\;\;\;\left(0\leq x^{*}\leq \frac{w}{\;L\;}\right)$$

(14) $$\frac{\partial \varphi \left(x^{*},1\right)}{\partial y^{*}}=0\;\;\;\;\;\;\;\left(\frac{w}{\;L\;}<x^{*}<1-\frac{w}{\;L\;}\right)$$

(15) $$\varphi \left(x^{*},1\right)=0\;\;\;\;\;\;\;\left(1-\frac{w}{\;L\;}\leq x^{*}\leq 1\right)$$

(16) $$\varphi \left(x^{*},0\right)=0$$

(17) $$\frac{\partial \varphi \left(0,y^{*}\right)}{\partial x^{*}}=0$$

(18) $$\frac{\partial \varphi \left(1,y^{*}\right)}{\partial x^{*}}=0$$

Equations (12)–(18) collectively form a mixed boundary value problem. It is particularly difficult to obtain a closed-form solution in the case of this analysis. Therefore, to obtain a solution by using the quadrature method, the Neumann boundary condition in Equation (14) was replaced by a Dirichlet boundary condition:

(19) $$\varphi \left(x^{*},1\right)=\frac{\;V_{\mathrm{rms}}\;}{\pi }\left[\frac{\pi }{\;2\;}-C\frac{x^{*}-\frac{1}{\;2\;}}{\;\frac{1}{\;2\;}-\frac{w}{\;L\;}\;}-\left(\frac{\pi }{\;2\;}-C\right)\frac{{\left(x^{*}-\frac{1}{\;2\;}\right)}^{3}}{\;6{\left(\frac{1}{\;2\;}-\frac{w}{\;L\;}\right)}^{3}\;}\right]\;\;\;\;\;\;\;\left(\frac{w}{\;L\;}<x^{*}<1-\frac{w}{\;L\;}\right).$$

Here, C is an arbitrary constant, and its optimal value was determined by the least squares method, yielding C=0.75480 (Appendix A).

### 2.3. Analytical Solution Using the Fourier Series Method

The functional form of $\varphi =\varphi \left(x^{*},y^{*}\right)$ is determined as follows, assuming $k_{n}=n\pi \left(n=1,\;2,\;\cdots \right)$ (Appendix B)

(20) $$\varphi =a_{0}y^{*}+2\overset{\infty }{\underset{n=1}{\sum }}a_{n}\cos\left(k_{n}x^{*}\right)\sinh\left(\frac{\;k_{n}H\;}{L}y^{*}\right)$$

(21) $$a_{0}=\frac{\;V_{\mathrm{rms}}\;}{2}$$

(22) $$\begin{array}{c} a_{n}=\frac{V_{\mathrm{rms}}}{k_{n}}\{\left(1-\frac{2C}{\pi }\right)-\frac{4C}{\pi k_{n}\frac{L-2w}{L}}\tan\left(\frac{k_{n}}{2}\frac{L-2w}{L}\right)\\ -\frac{24\left(1-\frac{2C}{\pi }\right)}{{k_{n}}^{2}{\left(\frac{L-2w}{L}\right)}^{3}}\{\frac{L-2w}{L}\left[\frac{{k_{n}}^{2}}{24}{\left(\frac{L-2w}{L}\right)}^{2}-1\right]\\ +\frac{2}{k_{n}}\left[\frac{{k_{n}}^{2}}{8}{\left(\frac{L-2w}{L}\right)}^{2}-1\right]\cot\left(\frac{k_{n}}{2}\right)\}\}\frac{\sin\left(\frac{k_{n}}{2}\right)\cos\left(\frac{k_{n}}{2}\frac{L-2w}{L}\right)}{\sinh\left(\frac{k_{n}H}{L}\right)} \end{array}$$

### 2.4. Analytical Form of DEP Force

Since the electric field $\;E=E\left(x^{*},y^{*}\right)$ is a vector quantity, it can be represented by the vector expression as

(23) $$\;E=E_{x}e_{x}+E_{y}e_{y}=-\mathrm{grad}\varphi$$

where $e_{x}$ and $e_{y}$ denote the unit vectors in the x and *y* directions, respectively, and $E_{x}=E_{x}\left(x^{*},y^{*}\right)$ and $E_{y}=E_{y}\left(x^{*},y^{*}\right)$ are the x and y components of the electric field E. Using Equations (20)–(22), $E_{x}$ and $E_{y}$ become

(24) $$E_{x}=2\overset{\infty }{\underset{n=1}{\sum }}a_{n}k_{n}\sin\left(k_{n}x^{*}\right)\sinh\left(\frac{\;k_{n}H\;}{L}y^{*}\right)\;\;\;\;\;\;\;\;\;\;\;\;\;\;$$

(25) $$E_{y}=\frac{\;V_{\mathrm{rms}}\;}{2}-\frac{\;2H\;}{L}\overset{\infty }{\underset{n=1}{\sum }}a_{n}k_{n}\cos\left(k_{n}x^{*}\right)\cos h\left(\frac{\;k_{n}H\;}{L}y^{*}\right).\;\;\;$$

To find $F_{\mathrm{DEP}}$, we can utilize the following mathematical relationship:

(26) $$\begin{array}{ll} \nabla {\left|E\right|}^{2} & =\nabla \left({E_{x}}^{2}+{E_{y}}^{2}\right)\\ & =2\left(E_{x}\frac{\;dE_{x}\;}{dx^{*}}+E_{y}\frac{\;dE_{y}\;}{dx^{*}}\right)e_{x}+2\left(E_{x}\frac{\;dE_{x}\;}{dy^{*}}+E_{y}\frac{\;dE_{y\;}}{dy^{*}}\right)e_{y} \end{array}$$

Equation (1) becomes

(27) FDEP=4π ϵl( dc 2)3Re(β)[(Ex dEx dx∗+Ey dEy dx∗)ex+(Ex dEx dy∗+Ey dEy dy∗)ey].

$F_{\mathrm{DEP}}$ can be found by substituting Equations (24) and (25) into Equation (27).

## 3. Experimental Method

### 3.1. Cell Sample Preparation

Live non-tumorigenic human mammary (MCF10A) cells were used as cell samples. A culture medium consisting of 500 mL DMEM/F12 (11320033, Gibco, Waltham, MA, USA), 20 ng/mL Human epidermal growth factor (E9644, ThermoFisher, Waltham, MA, USA), 0.5 μg/mL Hydrocortisone (H4001, Sigma Aldrich, St. Louis, MI, USA), 100 ng/mL Cholera toxin (C8052, Sigma Aldrich, St. Louis, MI, USA), 5% (*v*/*v*) Horse serum (16050122, ThermoFisher, Waltham, MA, USA), and 10 μg/mL Human insulin (I9278, Sigma Aldrich, St. Louis, MI, USA) was used for cell cultivation. The cultured cells were incubated in a 5% CO_2_ incubator for 2–3 days. Experiments were conducted using cells at passages P5–10.

### 3.2. Preparation of Cell Sample Solution

A cell sample solution was prepared by suspending cells in a 300 mM isotonic mannitol solution to achieve a cell density of $\phi =8.0\times 10^{5}$ cells/mL. For fluorescence imaging observations, cells were incubated with Calcein AM (C396, Dojindo; $\lambda_{\mathrm{ex}}$ = 490 nm, $\lambda_{\mathrm{em}}$ = 515 nm). Calcein AM was introduced into the cells by incubating them in a PBS(−) solution with a Calcein AM concentration of 20 µM for 30 min. The conductivity of the cell sample solution was adjusted to $\sigma_{f}=1.0\times 10^{-3}$ S/m using the culture medium.

### 3.3. Fabrication of Cell Separation Device

A cell separation device was fabricated using the counter-interdigitated electrodes, and the relationship between the distribution of cell clusters formed on the electrode surface and the DEP force distribution, as determined through analytical solutions, was examined.

**Figure 4:**
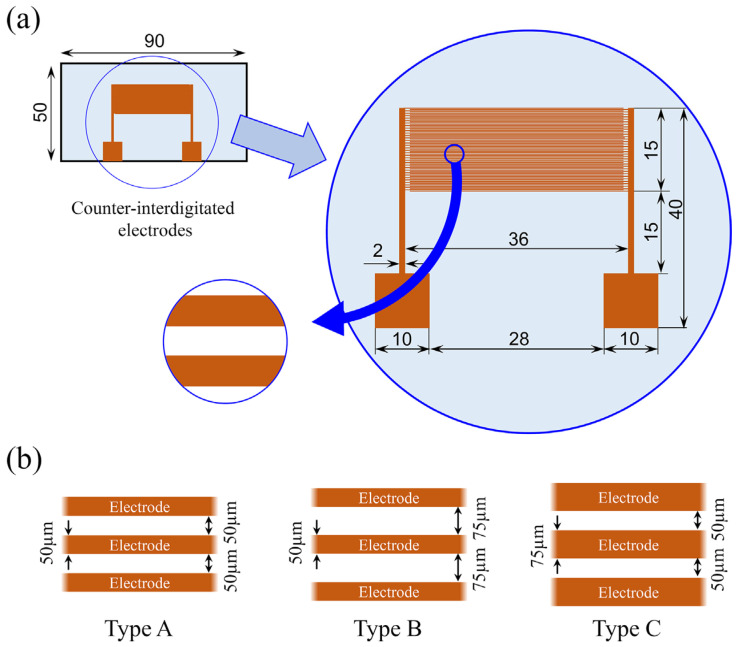
(**a**) Specifications of electrode substrate. (**b**) Configuration and dimensions of interdigitated electrodes.

Three types of interdigitated electrodes with different electrode widths and spacings were fabricated to generate different electric field distributions under the condition of a constant applied voltage. Figure 4a shows the dimensions of the electrode substrate used in the experiment, and Figure 4b shows the dimensions of the three types of interdigitated electrode. For the electrode width or spacing, combinations of 50 μm and 75 μm were considered. The interdigitated electrodes with (width:spacing) ratios were designated as Type A (50 μm–50 μm), Type B (50 μm–75 μm), and Type C (75 μm–50 μm). A standard photolithographic method was used to fabricate the interdigitated electrodes. The procedure is briefly described as follows. First, the surfaces of the glass planar plate with dimensions of 50×90 mm and a thickness of 0.4 mm were ultrasonically cleaned with acetone and isopropyl alcohol for 5 min each, followed by ultrapure water for 5 min. Next, a 300 nm thick aluminum film was deposited on the glass plate surface by vacuum evaporation. Electrode patterning was achieved using a photoresist (S1805G). Pre-baking was performed at 90 °C for 3 min, followed by a 20 s exposure and 1 min of development. Post-baking was performed at 130 °C for 3 min to fix the electrode to the glass plate surface. Then, etching treatment with mixed acid was performed at 40 °C for 90 s, and the photoresist was removed using an AZ 100 remover (AZ Electronic Materials). The fingers of the interdigitated electrodes were arranged in alternating fashion with high-voltage electrodes and grounded electrodes featuring 75 pairs for Type A and 60 pairs for Types B and C.

The cell separation device has a parallel-plate microfluidic channel structure with electrode substrates on the upper and lower surfaces, as shown in Figure 2. The upper electrode substrate is a 1.1 mm thick glass planar plate coated with an indium–tin oxide (ITO) film on its surface, while the lower electrode substrate is a glass planar plate printed with interdigitated electrodes. Holes with a diameter of ϕ1.0 mm were drilled in the upper electrode substrate using a leutor (2307396, Sea Force, Tokyo, Japan) for the introduction and drainage of the cell sample solution. Polypropylene female luer fittings were bonded to the drilled holes, and a conductive epoxy resin adhesive (CW2400, Chemtronics, Kennesaw, GA, USA) was used to attach lead wires to the terminals of each electrode substrate. Finally, a 0.5 mm thick silicone rubber spacer was placed between the upper and lower electrode substrates and compressed to complete the device’s assembly.

### 3.4. Experimental Apparatus and Method

**Figure 5:**
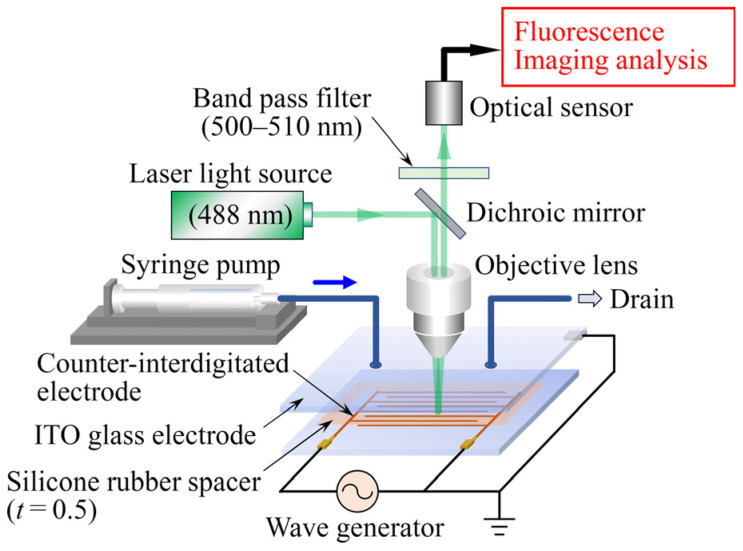
Schematic of the experimental setup.

The experimental setup consisted primarily of the cell separation device, a syringe pump, a waveform generator, and a confocal laser scanning microscope, as shown in Figure 5. To observe the behavior of cells in the device, the electric field frequency, f, that maximizes the value of Re(β) in Equation (1) was calculated using Equation (2) to maximize the DEP force acting on the cells. In the case of MCF10A cells, when the solution conductivity is $\sigma_{f}=1.0\times 10^{-3}$ S/m, the frequency that gives the maximum value of the Re(β) is f≅8.5 MHz. The value of Re(β) is Re(β)~0.98. From the above, the applied voltage was set as V=10 V_pp_ with *f* = 8.5 MHz.

The experimental procedure was as follows. First, 2 mL of the cell sample solution was introduced into the device using the syringe pump, and the flow was stopped after the degassing was confirmed. Next, the waveform generator was turned on to produce a nonuniform electric field in the device. After confirming the stability of cell distribution in the device through bright-field observation under the microscope, the z-scan function of the microscope was used to capture a series of slice fluorescence 2D images of the cell clusters. A 3D image was then constructed using the accompanying image analysis program to examine the distribution of cells adsorbed on the surface of the interdigitated electrodes.

## 4. Results and Discussion

### 4.1. Accuracy Verification of the Analytical Solution

**Figure 6:**
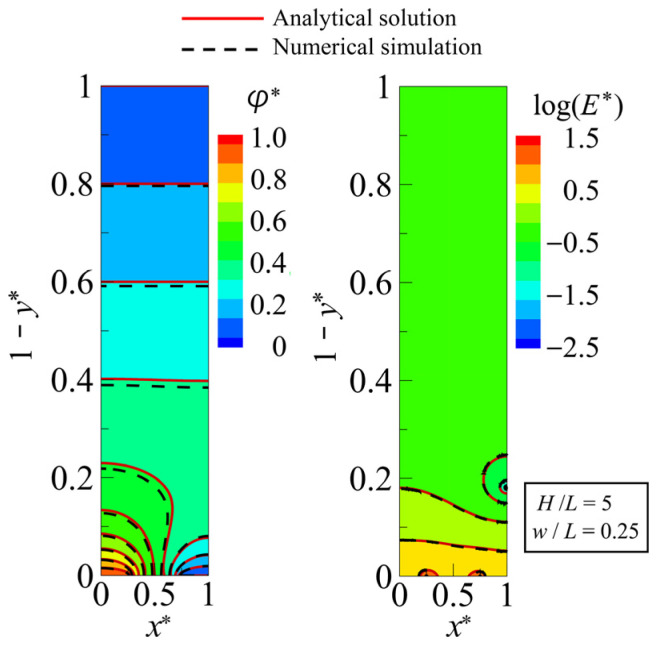
Comparison of analytical and numerical results for distributions of (**left**) electric potential and (**right**) electric field intensity.

**Figure 7:**
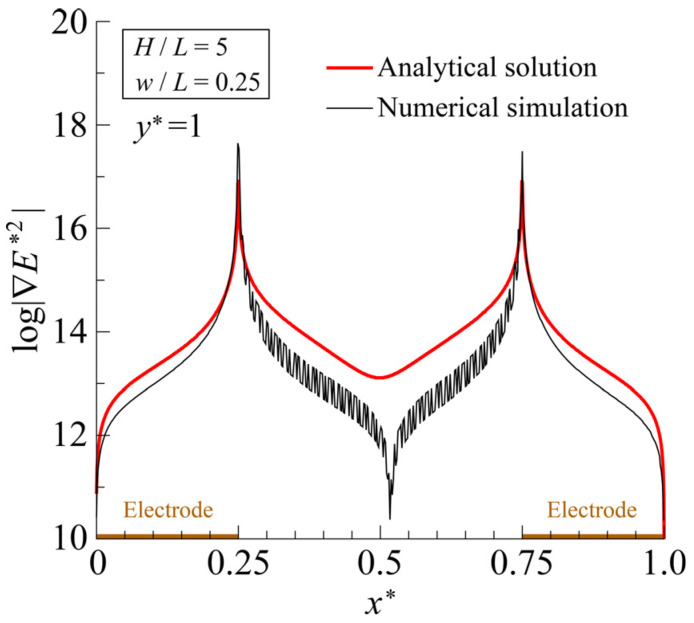
Distribution of $\left|\nabla {E^{*}}^{2}\right|$ on the interdigitated electrode surface $\left(y^{*}=1\right)$.

To verify the accuracy of the analytical solution obtained, a numerical simulation was performed using commercially available FEM software, FEATOOLS (https://www.featool.com, accessed on 16 April 2024), and the results were compared. The boundary conditions for the numerical simulation model were the same as those indicated in Figure 3. The numerical simulation was performed using approximately $2.6\times 10^{6}$ grid points and with a relative error of $1\times 10^{-6}$ for the convergence criterion of the iterative calculation. If the number of grid points exceeded $2.6\times 10^{6}$, the average relative error of the electric field distribution was less than 0.15% compared to, for example, results obtained with $1\times 10^{7}$ grid points, except for disparities in the electric field magnitude at the electrode edges $\left(x^{*}=w/L,1-2w/L\right)$. To compute the analytical solution, FORTRAN code was written in-house, and the number of terms in the series expansion, n, was set to n=10,000. The aspect ratios of the calculation domain were set to match those of the Type A device, at H/L=5 and w/L=0.25. For n>10,000, the error of the solution for n=20,000, for example, relative to that of n=10,000, was $\sim 3.0\times 10^{-10}$ %. Thus, n=10,000 gives sufficient accuracy for the solution. Figure 6 compares the analytical solution and numerical simulation for distributions of φ and E. The effective potential value, $V_{\mathrm{rms}}$, was set to $5/\sqrt{2}$ V, matching the applied voltage adopted in the experiment, and φ and E were each normalized to $\varphi /V_{\mathrm{rms}}$ and $EH/V_{\mathrm{rms}}$, respectively. For $\varphi^{*}$ and $\;E^{*}$ at the interdigitated electrode surface $\left(y^{*}=1\right)$, the average relative errors, $\Delta \varphi^{*}$ and $\Delta E^{*}$, between the numerical simulation and the analytical solutions were 6.24% and 6.29%, respectively. These results showed good agreement, affirming that the analytical solutions provided reasonable outcomes. Figure 7 compares the analytical solution and numerical simulation results for the distribution of $\left|\nabla {E^{*}}^{2}\right|$ along the bottom surface of the microfluidic channel. The analytical solution overestimated the magnitude of $\left|\nabla {E^{*}}^{2}\right|$ in the inter-electrode region $\left(0.25<x^{*}<0.75\right)$ by about 10% compared to the numerical simulation results. On the other hand, in the region from the electrode edge, where the value of $\left|\nabla {E^{*}}^{2}\right|$ is at its maximum, extending across the entire electrode surface, the two were in relatively good agreement. From Equation (1), the magnitude of the DEP force is proportional to that of the gradient of the square of electric field, written as $\left|F_{\mathrm{DEP}}\right|=F_{\mathrm{DEP}}\sim \left|\nabla E^{2}\right|$. Thus, the results demonstrated that the analytical solution of $\nabla {E^{*}}^{2}$ gives a reasonable distribution of $F_{\mathrm{DEP}}$. While examining the inter-electrode distribution of $\left|\nabla {E^{*}}^{2}\right|$ in the numerical simulation, despite utilizing a sufficient number of grid points ($2.6\times 10^{6}$), strong spatial oscillations occurred, preventing the correct distribution from being obtained. This occurs due to its proximity to the two electrode edges, which align with the mathematical singular points, making the numerical integration extremely unstable. For example, the numerical integration generally gives a spatially continuous solution for $F_{\mathrm{DEP}}$, a function of the second-order derivative of the potential, but it does not always guarantee the smooth continuity of the solution. The effect is particularly pronounced in regions where local values change rapidly, such as positions near the singular point. The solution either has strong spatial oscillations or diverges without convergence. Spurious oscillations in the distribution of $F_{\mathrm{DEP}}$ are inevitable even when other commercial FEM software is used [24,47]. On the other hand, the analytical solution is particularly useful for examining the force field in the DEP device, because it always gives a smooth and stable solution for physical quantities expressed in terms of the second- or higher-order derivative of φ, such as $\nabla E^{2}$.

### 4.2. Distribution of DEP Force

**Figure 8:**
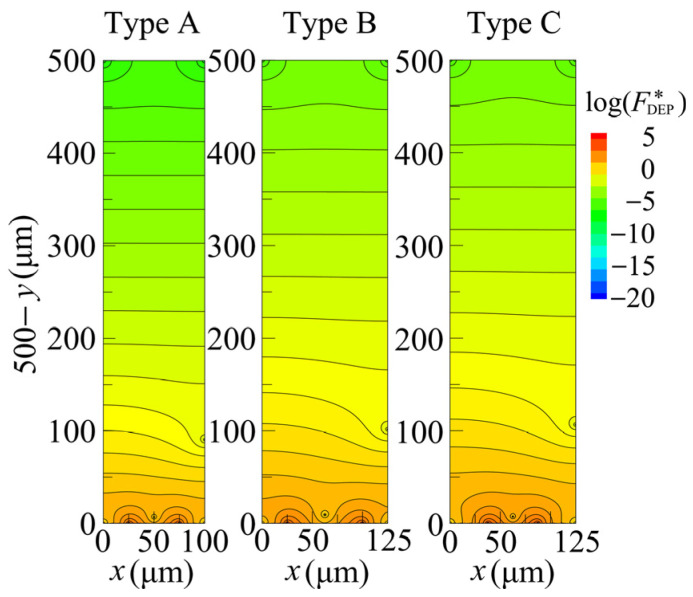
Distribution of DEP forces generated by three types of interdigitated electrodes.

Figure 8 shows the distributions of $F_{\mathrm{DEP}}\;\left(=\left|F_{\mathrm{DEP}}\right|\right)$ induced in cells by means of the three types of devices, Types A, B, and C. Note that $F_{\mathrm{DEP}}$ is normalized as ${F_{DEP}}^{*}=F_{DEP}/F_{R}$. The $F_{R}$ represents the magnitude of the DEP force, defined as FR=2π ϵl(dc/2)3Re(β) φ2/H3. The analysis conditions are listed in Table 1. For all types of interdigitated electrodes, an extremely strong force field was created around the electrode edges. The strength of ${F_{\mathrm{DEP}}}^{*}$ was significant at the electrode surface and hardly decayed from the electrode surface to a height of ~20 µm, which is equivalent to the size of one or two cells. The value of ${F_{\mathrm{DEP}}}^{*}$ began to decay rapidly as the distance from the electrode surface increased, occurring at an approximate height of over 20 µm. At a height of ~30 µm, the value of ${F_{\mathrm{DEP}}}^{*}$ decayed by two to three orders of magnitude. The ${F_{\mathrm{DEP}}}^{*}$ reached a minimum value at the midpoint between two adjacent electrodes because the $x^{*}$ axis components of ${F_{\mathrm{DEP}}}^{*}$ generated by these two electrodes acting on the cell at this position canceled each other out. Therefore, ${F_{\mathrm{DEP}}}^{*}$ is an even function with respect to the vertical axis passing through this center position, leading to ${F_{\mathrm{DEP}}}^{*}$ satisfying $\partial^{2}{F_{DEP}}^{*}/\partial x^{2}=0$. Figure 7 shows that the analytical solution satisfies this condition ($\partial^{2}\log\left(\nabla {E^{*}}^{2}\right)/\partial {x^{*}}^{2}=0$) at $x^{*}=0.5$, but the numerical simulation does not produce the correct distribution due to spatial oscillations.

### 4.3. Experimental Results

**Figure 9:**
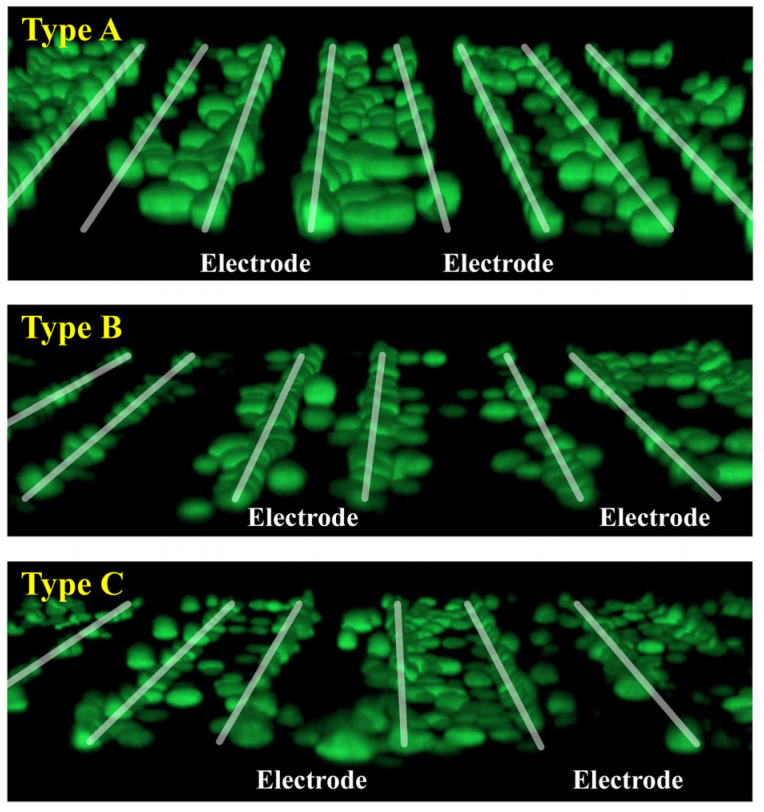
Three-dimensional fluorescent imaging of live cell distribution.

Figure 9 shows fluorescence images of the distribution of cells around the interdigitated electrodes of Types A, B, and C. For all three types, it can be seen that the cells were adsorbed along the electrode edges. This is because the $F_{\mathrm{DEP}}$ induced in the cells at each electrode edge was extremely large, and the cells that approached the electrode were attracted to this position first and then adsorbed. In addition, the low adsorption of cells to the central area of the electrode surface occurred because $F_{\mathrm{DEP}}$ was weaker there, as $E_{x}=0$. Comparing the distribution of $F_{\mathrm{DEP}}$ in Figure 8 with the distribution of cells in Figure 9, it can be seen that they are generally similar to each other. When Type A and Type C are compared, no significant difference in the cell distribution is found. This is likely due to the similarity in the $F_{\mathrm{DEP}}$ distribution within the inter-electrode region between the two types, as illustrated in Figure 8. Next, when comparing Types A and B, Type A showed cell adsorption in the inter-electrode region, while Type B showed almost no cell adsorption in the inter-electrode region. This is because, as shown in Figure 8, the value of $F_{\mathrm{DEP}}$ between electrodes was weaker in Type B than in Type A. As indicated above, cells tend to adsorb in the inter-electrode region rather than on the electrode surface, highlighting that the amount of cell adsorption, which determines the device’s cell separation performance, is affected more by adjusting the electrode spacing than by changing the electrode width.

### 4.4. Relationship Between DEP Force and Electrode Width and Spacing

**Figure 10:**
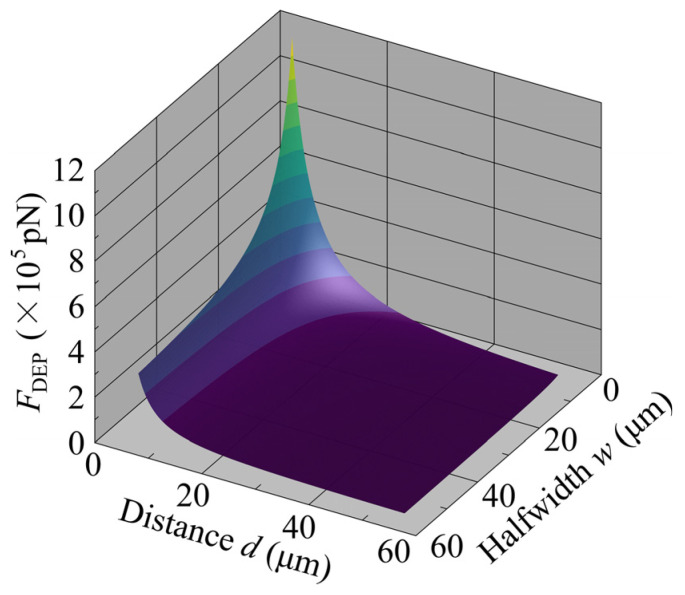
Variation of DEP force with variations in electrode width and spacing.

To investigate the effect of electrode architecture on DEP force, electrode width (2w) and electrode spacing,

(28) d=L−2w,

were used as variables in the parametric analysis. The physical properties used in this analysis are shown in Table 1. Figure 10 shows a bird’s-eye view of the change in the average value of $F_{DEP}$ on the interdigitated electrode surface as d and w were changed independently from 5 µm to 60 µm in 200–point increments. As the figure shows, the value of $F_{\mathrm{DEP}}$ changed more sensitively in response to changes in d than in response to changes in w. In addition, considering the connection between the distribution of $F_{\mathrm{DEP}}$ and that of w, it is thought that optimizing a device for highly efficient cell separation entails electrode spacing (d) of 30–50 µm. This design accounts for the sizes of the cells, particularly for cells of a size similar to MCF10A cells (*d_c_*~15 μm) used in this study. However, the details of the influence of this feature on the cell separation device performance require actual flow of cell samples at flow rates of 100–300 µm/s [10,42,48] and the evaluation of the cell separation ratio.

## 5. Conclusions

To investigate the distributions of the electric field intensity and DEP force within the newly proposed parallel-plate cell separation device, we obtained analytical solutions using the Fourier series method. By extrapolating the inter-electrode potential with a newly proposed approximation, the boundary condition, initially given in Neumann type, was replaced by Dirichlet type. This adjustment allowed for the integration of the governing equations to derive an approximate solution. To verify the accuracy of the obtained solution, a comparison with numerical simulations was performed, demonstrating excellent agreement for both the potential and electric field intensity distributions. In addition, 3D fluorescence imaging analysis of the cell distribution in the cell separation device was performed. The distribution of live cells near the interdigitated electrode surface was compared with that of the DEP force obtained by the analytical solution. This comparison revealed that the distribution of live cells was very similar to that of the DEP forces. In addition, a parametric analysis involving the electrode width and spacing as variables revealed for the first time that electrode spacing was the key parameter influencing the level of cell adsorption onto the electrode. In cell separation processes utilizing devices with interdigitated electrodes, electrode width is not very important for highly efficient cell separation. For example, when separating human cells with a diameter of about 15 μm, electrode spacing of 30–50 μm is desirable. On the other hand, many new devices specific for the separation and detection of rare cells such as CTCs have been proposed in recent years. The arrangement and shape of interdigitated electrodes implemented in those devices are often different from the electrodes analyzed in this study [2,3,4]. However, the knowledge obtained in this analysis regarding the optimization of electrode width and electrode spacing with respect to interdigitated electrodes may be useful in evaluating the performance of even devices with different types of electrode geometries. Additionally, in technology used to separate biological particles using DEP, alongside cells and DNA, bacterial detection and separation devices have been reported in many existing cases. For example, interdigitation electrode devices using DEP have been shown by Bisceglia, E. et al. [49] to be usable in detecting bacteria in human blood samples spiked with bacteria. In this report, optimization calculations were performed using the device’s channel height and electrode spacing as parameters. Therefore, optimization of the electrode arrangement and architecture based on electric field analysis is expected to contribute to improving the precision of bacterial separation and detection technologies in blood and body fluid samples. Furthermore, by placing the interdigitated electrodes on the bottom surface of a micro-well chip for bacterial detection, DEP can be generated within micro-scale wells, allowing for efficient bacterial aggregation. This is expected to enable the realization of more precise and rapid bacterial detection technologies. We expect that DEP will be integrated with bacterial testing systems in the future [50,51].

## Figures and Tables

**Table 1:** Analysis conditions.

Symbol		Values
RMS voltage $V_{\mathrm{rms}}$		$5/\sqrt{2}$ V
electric permittivity of the solution $\epsilon_{l}$		$6.9\times 10^{-10}$ F/m
electric conductivity of the solution $\sigma_{l}$		$1.0\times 10^{-3}$ S/m
cell diameter $d_{c}$		15 μm
height of the microchannel H		500 μm
real part of the CM factor Re(β)		0.98
number of terms of the series expansion n		100,000
electrode width and spacing (2w:*d*)	Type A	(50 μm:50 μm)
	Type B	(50 μm:75 μm)
	Type C	(75 μm:50 μm)

**Figure A1:**
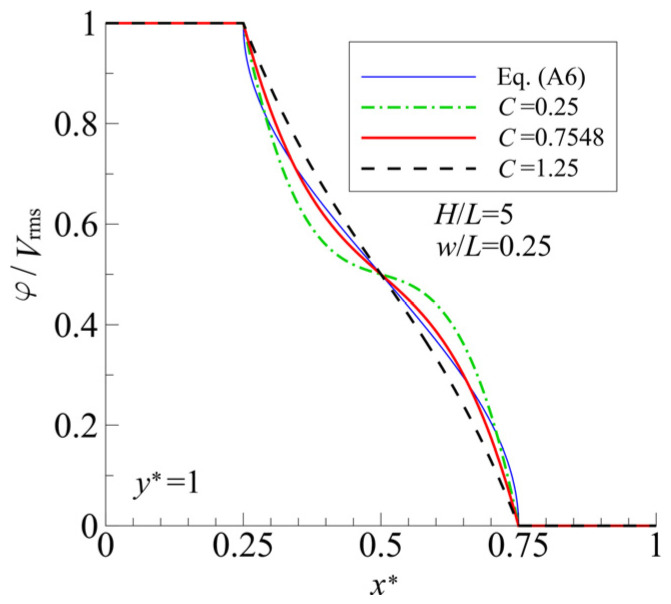
Distribution of $\varphi \left(=V\left(x^{*}\right)\right)$ for various values of C.

**Figure A2:**
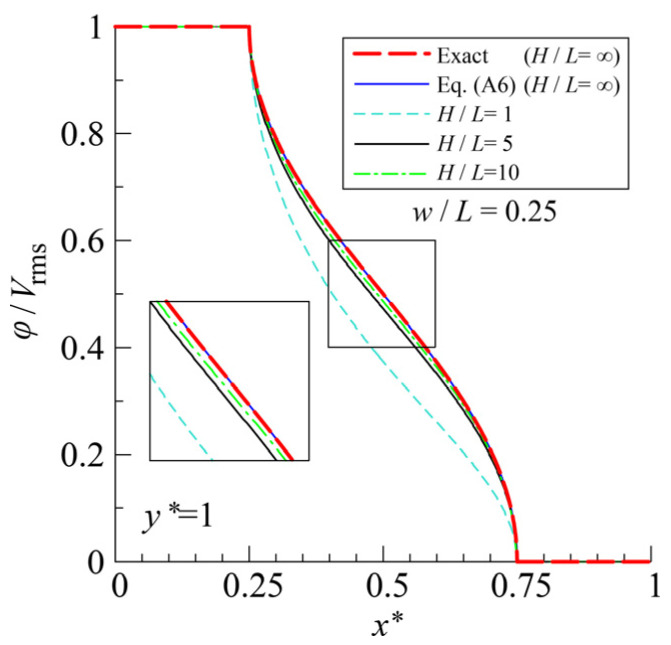
Inter-electrode potential distribution.

## Data Availability

Data will be provided on suitable request.

## Appendix A

The exact solution for the inter-electrode potential in the absence of the planar electrode on the top surface $\left(y^{*}=0\right)$ is expressed by an incomplete elliptic integral of the first kind with an elliptical modulus k(=(1−2w)⁄L) [35]:

(A1) $$F\left(\lambda ,k\right)=\int_{0}^{\lambda }\frac{d\theta }{\;\sqrt{1-k^{2}\sin^{2}\theta }\;}$$

Since the integration of F(λ,k) using the quadrature method is difficult, we will seek an approximate solution in integrable form. The function F(λ,k) can be expressed by using the inverse function of Jacobi’s elliptic function, Sn, as

(A2) $$F(\lambda ,k)={\mathrm{Sn}}^{-1}(\sin\lambda ,k)$$

If $\lambda =\sin^{-1}x^{\prime }$, then for k→0, i.e., 1−2d/L→0, we have

(A3) $$\mathrm{Sn}\left(x^{\prime },\;0\right)≅\sin\;x^{\prime }$$

Therefore, Equation (A2) can be approximated as

(A4) $$F\left(\lambda ,k\right)≅\sin^{-1}x^{\prime }$$

Here, the Neumann boundary condition in Equation (14) is replaced by a Dirichlet boundary condition:

(A5) $$\varphi \left(x^{*},1\right)=V\left(x^{*}\right)\;\;\;\;\;\;\;\left(\frac{w}{\;L\;}<x^{*}<1-\frac{w}{\;L\;}\right).$$

For an extrapolating function of $V\left(x^{*}\right)$ on the right-hand side of Equation (A5), the expression

(A6) $$V\left(x^{*}\right)=\frac{V_{\mathrm{rms}}}{\pi }\cos^{-1}x^{*}$$

was chosen. Equation (A6) is obtained by performing the variable transformation $x^{\prime }\to x^{*}$ in Equation (A4) and applying the appropriate similarity transformation to satisfy the boundary conditions of Equations (13) and (15). Equation (A6) closely approximates the exact solution for the inter-electrode potential in the absence of a planar electrode on the top surface of the device ($y^{*}=0$). To make Equation (A6) integrable in the Fourier series method, the third-order terms of the McLaurin expansion of $\cos^{-1}x^{*}$ were considered. This was carried out under conditions in which 1−2w/L≅0, resulting in the reduction of Equation (A6) to

(A7) $$V\left(x^{*}\right)⋍\frac{\;V_{\mathrm{rms}}\;}{\pi }\left[\frac{\pi }{\;2\;}-x^{*}-\frac{\;{\left(x^{*}\right)}^{3}\;}{6}\right]+O\left({\left(x^{*}\right)}^{5}\right).$$

Equation (A7), satisfying boundary conditions, $V\left(w/L\right)=V_{\mathrm{rms}}\;\mathrm{and}\;V\left(1-w/L\right)=0$, was obtained as follows:

(A8) $$V\left(x^{*}\right)=\frac{\;V_{\mathrm{rms}}\;}{\pi }\left[\frac{\pi }{\;2\;}-C\frac{x^{*}-\frac{1}{\;2\;}}{\;\frac{1}{\;2\;}-\frac{w}{\;L\;}\;}-\left(\frac{\pi }{\;2\;}-C\right)\frac{{\left(x^{*}-\frac{1}{\;2\;}\right)}^{3}}{\;6{\left(\frac{1}{\;2\;}-\frac{w}{\;L\;}\right)}^{3}\;}\right].$$

Here, C is an arbitrary constant, and its optimal value was determined by curve fitting Equation (A8) to Equation (A6) using the least squares method, yielding C=0.75480. Figure A1 shows the distribution of Equation (A8) normalized to the value $V_{\mathrm{rms}}$ for various values of C when H/L=5 and w/L=0.25, together with the distribution of normalized Equation (A6).

To evaluate the accuracy of Equation (A6), the inter-electrode potential obtained using it was compared with numerical simulation results obtained using commercial FEM software. The distribution of $\varphi =\varphi \left(x^{*},1\right)$ obtained from the exact solution of Equation (A2) and that obtained from Equation (A6) are shown in Figure A2. For comparison, distributions of φ obtained by numerical simulations with three different aspect ratios, H/L, are also shown in Figure 3. The results of the exact solution and Equation (A6) show remarkable agreement. Equation (A6) gives a good approximation of the inter-electrode potential when H/L>5, exhibiting relative errors within 5% of the commercial FEM software results.

## Appendix B

When the Laplace equation, Equation (3), is solved analytically through the separation-of-variables technique, the general form of the solution $\varphi =\varphi \left(x^{*},y^{*}\right)$ is expressed as

(A9) $$\varphi =A^{\prime }+B^{\prime }x^{*}+C^{\prime }y^{*}+D^{\prime }x^{*}y^{*}+f\left(x^{*}\right)g\left(y^{*}\right)$$

where $A^{\prime }$, $B^{\prime }$, $C^{\prime }$, and $D^{\prime }$ are unknown constants, and f and g are functions of only $x^{*}$ and $y^{*}$ satisfying Equations (13)–(18), respectively. Substituting Equation (16) into Equation (A9) yields

(A10) $$A^{\prime }=B^{\prime }=0$$

Furthermore, substitution of Equations (17) and (18) into Equation (A9) yields

(A11) $$D^{\prime }=0.$$

The functional form of $f\left(x^{*}\right)g\left(y^{*}\right)$ on the right-hand side of Equation (A9) is expressed as

(A12) $$\;f\left(x^{*}\right)g\left(y^{*}\right)=\overset{\infty }{\underset{n=1}{\sum }}\left(a_{n}\cos k_{n}x^{*}+b_{n}\sin k_{n}x^{*}\right)(c_{n}e^{k_{n}y^{*}}+d_{n}e^{-k_{n}y^{*}})$$

where $a_{n},b_{n},c_{n},d_{n}$ and $k_{n}$ are arbitrary coefficients. From Equations (17) and (18),

(A13) $$b_{n}=0$$

(A14)
$$k_{n}=n\pi$$

Furthermore, from Equation (16),

(A15) $$c_{n}=-d_{n},$$

and therefore, Equation (A12) becomes

(A16) $$f\left(x^{*}\right)g\left(y^{*}\right)=2\overset{\infty }{\underset{n=1}{\sum }}a_{n}\cos\left(k_{n}x^{*}\right)\sinh\left(\frac{k_{n}H}{L}y^{*}\right)\;$$

Using Equations (13), (15), (A5) and (A8), the functional form of $\varphi \left(x^{*},y^{*}\right)$ is determined as

(A17) $$\varphi \left(x^{*},y^{*}\right)=a_{0}y^{*}+2\overset{\infty }{\underset{n=1}{\sum }}a_{n}\cos\left(k_{n}x^{*}\right)\sinh\left(\frac{k_{n}H}{L}y^{*}\right)$$

where $a_{0}$ and $a_{n}$ are the Fourier coefficients. Because $\varphi \left(x^{*},1\right)$ is a periodic function of period $x^{*}=1$, the Fourier coefficient $a_{0}$ can be expressed as

(A18) $$a_{0}=\int_{0}^{1}\varphi \left(x^{*},1\right)dx^{*}\;$$

Because the right-hand side of Equation (A18) is equal to the area of the figure bounded by the curve of potential $\varphi \left(x^{*},1\right)$ and the $x^{*}$ and $y^{*}$ axes shown in Figure A1, $a_{0}$ can be readily obtained as

(A19) $$a_{0}=V_{\mathrm{rms}}\frac{w}{\;L\;}+\frac{1}{\;2\;}V_{\mathrm{rms}}\left(1-\frac{w}{\;L\;}-\frac{w}{\;L\;}\right)=\frac{\;V_{\mathrm{rms}}\;}{2}$$

For $a_{n}$, if $y^{*}=1$ in Equation (A17), further multiplying both sides by $\cos k_{m}x^{*}$ and integrating over the interval [0,1] yields

(A20) $$\int_{0}^{1}\varphi \left(x^{*},1\right)\cos\left(k_{m}x^{*}\right)dx^{*}=\int_{0}^{1}a_{0}\cos\left(k_{m}x^{*}\right)dx^{*}+2\int_{0}^{1}\overset{\infty }{\underset{n=1}{\sum }}a_{n}\cos\left(k_{n}x^{*}\right)\sinh\left(\frac{k_{n}H}{L}\right)\cos\left(k_{m}x^{*}\right)dx^{*}$$

Due to the periodicity and orthogonality of trigonometric functions,

$$\int_{0}^{1}a_{0}\cos\left(k_{n}x^{*}\right)dx^{*}=0$$

(A21)
$$\begin{array}{c} 2\int_{0}^{1}\overset{\infty }{\underset{n=1}{\sum }}a_{n}\cos\left(k_{n}x^{*}\right)\sinh\left(\frac{k_{n}H}{L}\right)\cos\left(k_{m}x^{*}\right)dx^{*}\\ =2a_{n}\sinh\left(\frac{k_{n}H}{L}\right)\int_{0}^{1}\cos^{2}\left(k_{n}x^{*}\right)dx^{*}\\ =a_{n}\sinh\left(\frac{k_{n}H}{L}\right)\;\# \end{array}$$

Since it is required that $a_{n}$ is

(A22) $$a_{n}=\frac{1}{\;\sin h\left(\frac{k_{n}H}{L}\right)\;}\int_{0}^{1}\varphi \left(x^{*},1\right)\cos\left(k_{n}x^{*}\right)dx^{*}$$

substituting Equations (13), (15), (A5) and (A8) into Equation (A22) yields Equation (22).

## References

[1] Farasat M., Chavoshi S.M., Bakhshi A., Valipour A., Badieirostami M. (2021). A dielectrophoresis-based microfluidic chip for trapping circulating tumor cells using a porous membrane. J. Micromech. Microeng..

[2] Lv B., Cai J. (2023). Simulation and analysis of geometric parameters based on Taguchi method in YY microfluidic device for circulating tumor cell separation by alternating current dielectrophoresis. J. Chromatogr. A.

[3] Valijam S., Salehi A., Andersson M. (2023). Design of a low-voltage dielectrophoresis lab-on-the chip to separate tumor and blood cells. Microfluid. Nanofluid..

[4] Varmazyari V., Habibiyan H., Ghafoorifard H., Ebrahimi M., Ghafouri-Fard S. (2022). A dielectrophoresis-based microfluidic system having double-sided optimized 3D electrodes for label-free cancer cell separation with preserving cell viability. Sci. Rep..

[5] Giduthuri A.T., Theodossiou S.K., Schiele N.R., Srivastava S.K. (2020). Dielectrophoresis as a tool for electrophysiological characterization of stem cells. Biophys. Rev..

[6] Sarno B., Heineck D., Heller M.J., Ibsen S.D. (2021). Dielectrophoresis: Developments and applications from 2010 to 2020. Electrophoresis.

[7] Boran Z., Fan Y., Wenshuai W., Wuyi W., Wenhan Z., Qianbin Z. (2022). Investigation of particle manipulation mechanism and size sorting strategy in a double-layered microchannel. Lab. Chip.

[8] Ni C., Wu D., Chen Y., Wang S., Xiang N. (2024). Cascaded elasto-inertial separation of malignant tumor cells from untreated malignant pleural and peritoneal effusions. Lab. Chip.

[9] Abidin Z.Z., Downes L., Markx G.H. (2007). Novel electrode structures for large scale dielectrophoretic separations based on textile technology. J. Biotechnol..

[10] Huang Y., Wang X.B., Becker F.F., Gascoyne P.R.C. (1997). Introducing dielectrophoresis as a new force field for field-flow fractionation. Biophys. J..

[11] Fiedler S., Shirley S.G., Schnelle T., Fuhr G. (1998). Dielectrophoretic sorting of particles and cells in a microsystem. Anal. Chem..

[12] Morgan H., Izquierdo A.G., Bakewell D., Green N.G., Ramos A. (2001). The dielectrophoretic and travelling wave forces generated by interdigitated electrode arrays: Analytical solution using Fourier series. J. Phys. D Appl. Phys..

[13] Choi S., Park J.K. (2005). Microfluidic system for dielectrophoretic separation based on a trapezoidal electrode array. Lab. Chip.

[14] Song H., Bennett D.J. (2010). A semi-analytical approach using artificial neural network for dielectrophoresis generated by parallel electrodes. J. Electrost..

[15] Lewpiriyawong N., Yang C., Lam Y.C. (2010). Continuous sorting and separation of microparticles by size using AC dielectrophoresis in a PDMS microfluidic device with 3-D conducting PDMS composite electrodes. Electrophoresis.

[16] So J.H., Dickey M.D. (2011). Inherently aligned microfluidic electrodes composed of liquid metal. Lab. Chip.

[17] Fathy J., Pourmand A., Ghavifekr H.B. (2017). Design and simulation of a MEMS based cell separator utilizing 3D travelling-wave dielectrophoresis. Microsyst. Technol..

[18] Jiang T., Ren Y., Liu W., Tang D., Tao Y., Xue R., Jiang H. (2018). Dielectrophoretic separation with a floating-electrode array embedded in microfabricated fluidic networks. Phys. Fluids.

[19] Beech J.P., Keim K., Ho B.D., Guiducci C., Tegenfeldt J.O. (2019). Active posts in deterministic lateral displacement devices. Adv. Mater. Technol..

[20] Alnaimat F., Mathew B., Hilal-Alnaqbi A. (2020). Modeling a dielectrophoretic microfluidic device with vertical interdigitated transducer electrodes for separation of microparticles based on size. Micromachines.

[21] Huang X., Torres-Castro K., Varhue W., Salahi A., Rasin A., Honrado C., Brown A., Guler J., Swami N.S. (2021). Self-aligned sequential lateral field non-uniformities over channel depth for high throughput dielectrophoretic cell deflection. Lab. Chip.

[22] Tada S., Seki Y. (2022). Analysis of temperature field in the dielectrophoresis-based microfluidic cell separation device. Fluids.

[23] Zhang J., Song Z., Liu Q., Song Y. (2020). Recent advances in dielectrophoresis-based cell viability assessment. Electrophoresis.

[24] Nerguizian V., Alazzam A., Roman D., Stiharu I., Burnier M. (2012). Analytical solutions and validation of electric field and dielectrophoretic force in a bio-microfluidic channel. Electrophoresis.

[25] Alazzam A., Roman D., Nerguizian V., Stiharu I., Bhat R. (2010). Analytical formulation of electric field and dielectrophoretic force for moving dielectrophoresis using Fourier series. Microfluid. Nanofluid.

[26] Chen R., Liu R., Shen H. Modeling and analysis of electric field and electrostatic adhesion force generated by interdigital electrodes for wall climbing robots. Proceedings of the IEEE/RSJ International Conference on Intelligent Robots and Systems.

[27] Alhammadi F., Waheed W., El-Khasawneh B., Alazzam A. Mathematical model and verification of electric field and dielectrophoresis in a microfluidic device. Proceedings of the 2018 Advances in Science and Engineering Technology International Conferences (ASET).

[28] Clague D., Wheeler E. (2001). Dielectrophoretic manipulation of macromolecules: The electric field. Phys. Rev. E.

[29] Gurtner M., Hengster-Movric K., Hurák Z. (2017). Green’s function-based control-oriented modeling of electric field for dielectrophoresis. J. Appl. Phys..

[30] Garcia M., Clague D. (2000). The 2D electric field above a planar sequence of independent strip electrodes. J. Phys. D Appl. Phys..

[31] Wang X., Wang X.B., Becker F., Gascoyne P.R.C. (1996). A theoretical method of electrical field analysis for dielectrophoretic electrode arrays using Green’s theorem. J. Phys. D Appl. Phys..

[32] Bai S., Tang Y., Ruan L., Song R., Chen H., Du Y., Lin H., Tang Y., Shan Y. (2023). Investigation into the influence of interdigital parameters on electrochemical performance for in-plane supercapacitors via mathematical modeling and conformal mapping techniques. J. Energy Storage.

[33] Blume S.O., Ben-Mrad R., Sullivan P.E. (2015). Modelling the capacitance of multi-layer conductor-facing interdigitated electrode structures. Sens. Actuators B Chem..

[34] Pampin R.S., Raskin J., Huynen I., Flandre D. (2020). Electrodes-oxide-semiconductor device for biosensing: Renewed conformal analysis and multilayer algorithm. J. Electroanal. Chem..

[35] Sun T., Green N.G., Gawad S., Morgan H. (2007). Analytical electric field and sensitivity analysis for two microfluidic impedance cytometer designs. IET Nanobiotechnol..

[36] Sun T., Morgan H., Green N.G. (2007). Analytical solutions of ac electrokinetics in interdigitated electrode arrays: Electric field, dielectrophoretic and traveling-wave dielectrophoretic forces. Phys. Rev. E.

[37] Sharbati P., Elitas M. Numerical analysis of microfluidic gold electrode array for dielectrophoretic characterization of U87 glioma cells. Proceedings of the 2022 Medical Technologies Congress.

[38] Ghomian T., Hihath J. (2022). Review of dielectrophoretic manipulation of micro and nanomaterials: Fundamentals, recent developments, and challenges. IEEE Trans. Biomed. Eng..

[39] Miura T., Uno S. (2019). Computer simulation for electrochemical impedance of a living cell adhered on the inter-digitated electrode sensors. Jpn. J. Appl. Phys..

[40] Santos-Neto I.S.d., Carvalho C.D., Filho G.B.A., Andrade C.D.S.S., Santos G.C.d.O., Barros A.K., Neto J.V.d.F., Casas V.L.P., Alencar L.M.R., Lopes A.J.O. (2021). Interdigitated electrode for electrical characterization of commercial pseudo-binary biodiesel–diesel blends. Sensors.

[41] Zaman M.A., Padhy P., Ren W., Wu M., Hesselink L. (2021). Microparticle transport along a planar electrode array using moving dielectrophoresis. J. Appl. Phys..

[42] Tada S., Hayashi M., Eguchi M., Tsukamoto A. (2017). High-hroughput separation of cells by dielectrophoresis enhanced with 3D gradient AC electric field. Biomicrofluidics.

[43] Duffy D.G. (2008). Mixed Boundary Value Problems.

[44] Feng J.J., Krishnamoorthy S., Chen Z.J., Makhijani V.B. Numerical and analytical studies of AC electric field in dielectrophoretic electrode arrays. Proceedings of the Technical Proceedings of the 2002 International Conference on Modeling and Simulation of Microsystems.

[45] Jones T.B. (1995). Electromechanics of Particles.

[46] Henslee E.A., Sano M.B., Rojas A.D., Schmelz E.M., Davalos R.V. (2011). Selective concentration of human cancer cells using contactless dielectrophoresis. Electrophoresis.

[47] Gauthier V., Bolopion A., Gauthier M. (2017). Analytical formulation of the electric field induced by electrode arrays: Towards automated dielectrophoretic cell sorting. Micromachines.

[48] Afshin S., Morteza B., Afshin A.N. (2023). Electrode-based dielectrophoretic separation of live and dead yeast cells. Iran. J. Chem. Chem. Eng..

[49] Bisceglia E., Cubizolles M., Mallard F., Pineda F., Francais O., Le Pioufle B. Optimization of dielectrophoretic separation and concentration of pathogens in complex biological samples. Proceedings of the SPIE 8765, Bio-MEMS and Medical Microdevices.

[50] Yin W., Hu K., Yu B., Zhang T., Mei H., Zhang B., Zou Z., Xia L., Gui Y., Yin J. (2024). Fast and sensitive detection of viable *Escherichia coli* O157:H7 using a microwell-confined and propidium monoazide-assisted digital CRISPR microfluidic platform. Lab. Chip.

[51] Hsieh K., Zec H.C., Chen L., Kaushik A.M., Mach K.E., Liao J.C., Wang T. (2018). Simple and precise counting of viable bacteria by resazurin-amplified picoarray detection. Anal. Chem..

